# New cytotoxic indole derivatives with anti-FADU potential produced by the endophytic fungus *Penicillium oxalicum* 2021CDF-3 through the OSMAC strategy

**DOI:** 10.3389/fmicb.2024.1400803

**Published:** 2024-05-30

**Authors:** Wei Song, Lianlian Ji, Yanxia Zhang, Longhe Cao

**Affiliations:** ^1^Department of Otolaryngology, The Third Affiliated Hospital of Wenzhou Medical University, Zhejiang, China; ^2^Department of Pediatrics, The Third Affiliated Hospital of Wenzhou Medical University, Zhejiang, China; ^3^Shandong Research Center of Engineering and Technology for Safety Inspection of Food and Drug, Shandong Institute for Food and Drug Control, Jinan, China

**Keywords:** *Penicillium oxalicum*, fungal secondary metabolites, indole derivatives, OSMAC method, cytotoxicity

## Abstract

Fungi possess well-developed secondary metabolism pathways that are worthy of in-depth exploration. The One Strain Many Compounds (OSMAC) strategy is a useful method for exploring chemically diverse secondary metabolites. In this study, continued chemical investigations of the marine red algae-derived endophytic fungus *Penicillium oxalicum* 2021CDF-3 cultured in PDB media yielded six structurally diverse indole derivatives, including two new prenylated indole alkaloids asperinamide B (**1**) and peniochroloid B (**5**), as well as four related derivatives (compounds **2**–**4** and **6**). The chemical structures of these compounds, including the absolute configurations of **1** and **5**, were determined by extensive analyses of HRESIMS, 1D and 2D NMR spectroscopic data, and TDDFT-ECD calculations. Compound **1** was found to possess an unusual 3-pyrrolidone dimethylbenzopyran fused to the bicyclo[2.2.2]diazaoctane moiety, which was rare in previously reported prenylated indole alkaloids. *In vitro* cytotoxic experiments against four human tumor cell lines (HeLa, HepG2, FADU, and A549) indicated that **1** strongly inhibited the FADU cell line, with an IC_50_ value of 0.43 ± 0.03 μM. This study suggested that the new prenylated indole alkaloid **1** is a potential lead compound for anti-FADU drugs.

## 1 Introduction

Secondary metabolism in filamentous fungi is well-developed (Gerke and Braus, 2014). These organisms are prolific producers of structurally diverse secondary metabolites that exhibit various promising biological properties (Zhang et al., 2019; Li et al., 2021). In the study of natural products, many useful methods, including genetic and cultivation-based strategies, have been developed to activate silent or cryptic secondary metabolites (Yuan et al., 2020a; Pinedo-Rivilla et al., 2022). Among them, the One Strain Many Compounds (OSMAC) approach, which is conceptualized as a single strain that can produce different metabolites when cultured under different conditions, is among the most effective tools for regulating microbial secondary metabolism (Romano et al., 2018; Ying et al., 2018).

Indole alkaloids have a bicyclic structure that consists of a six-membered benzene ring fused to a five-membered nitrogen-containing pyrole ring (Hu et al., 2021; Umer et al., 2022). Indole alkaloids are among the most important secondary metabolites for drug developments (Zhang et al., 2020). Prenylated indole alkaloids with a bicyclo[2.2.2]diazaoctane ring system are well known for their chemical, biosynthetic, and biological interests (Zhao et al., 2023). Structurally, prenylated indole alkaloids contain a bicyclo[2.2.2]diazaoctane framework and densely functionalized indole-derived subunits. These alkaloids represent a large and highly structurally diverse group of secondary metabolites that exhibit numerous potent pharmaceutical properties (Zhang et al., 2019). It has been reported that prenylated indole alkaloids possess anticancer, antimalarial, antimicrobial, anti-inflammatory, anti-diabetic, and immune-regulatory activities (Zhang et al., 2019; Zhao et al., 2023).

**Figure 1:**
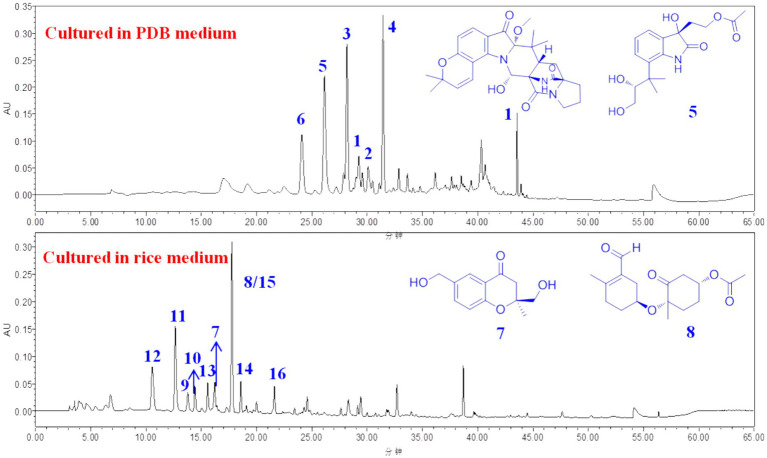
HPLC profile of crude extracts of *P. oxalicum* 2021CDF-3 in different cultural conditions.

**Figure 2:**
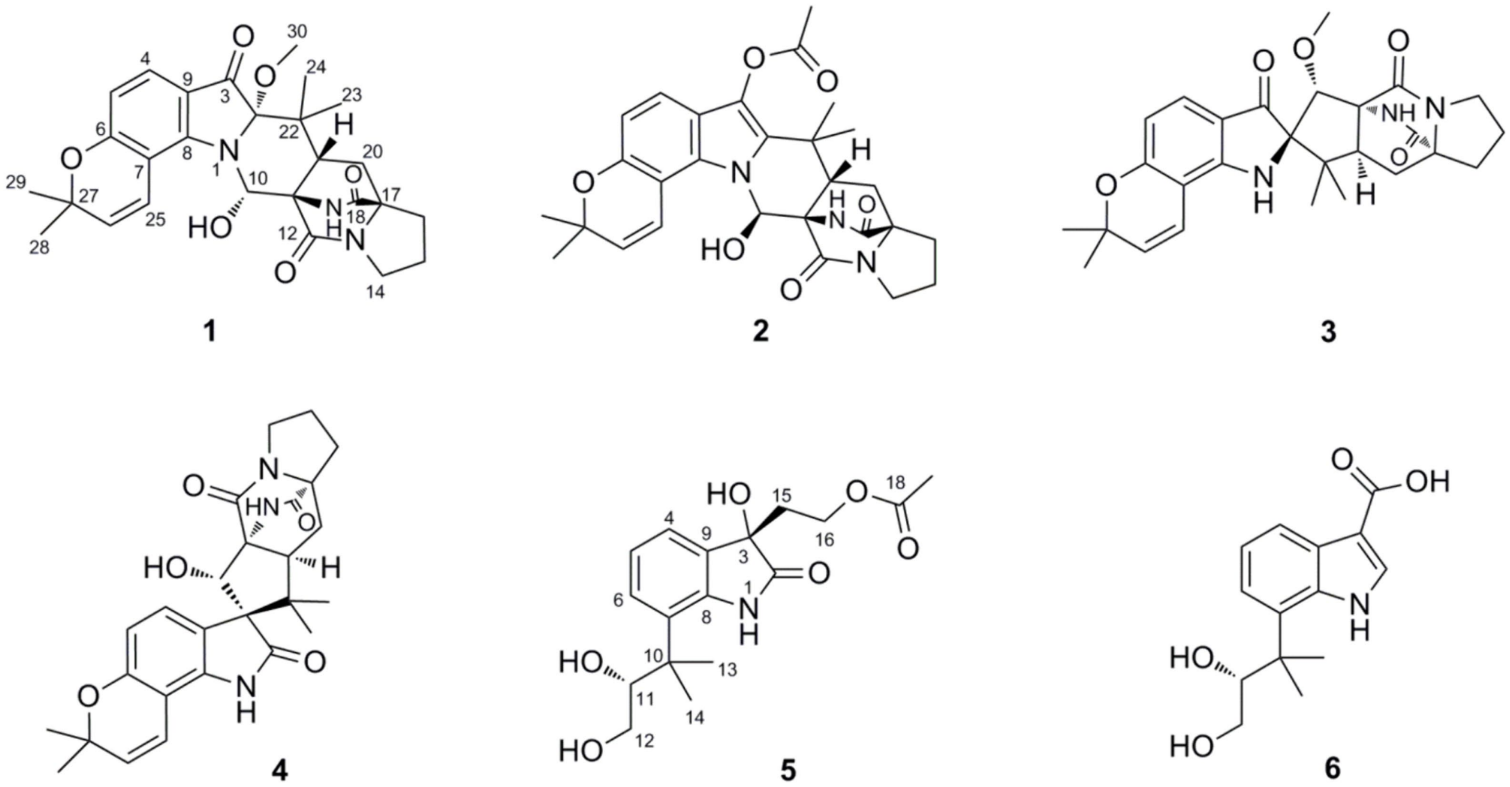
Structures of the isolated compounds **1**–**6.**

The filamentous fungus *Penicillium oxalicum* is a patented biocontrol and industrial producing strain that is used to prepare biological pesticides and degrading enzymes (Tian et al., 2018). Compared with those of other species in the *Penicillium* genus (Zhang et al., 2020), the secondary metabolites of *P. oxalicum* have not been extensively studied, and only a limited family of metabolites, such as phenalenone derivatives (Qi et al., 2023), dihydrothiophene-condensed chromones (Sun et al., 2012), decaturin alkaloids (Wang et al., 2013), and phenolic enamides and meroterpenoids (Li et al., 2015), has been reported. The fungus *P. oxalicum* 2021CDF-3 used in this study was previously isolated from the inner tissue of the marine red alga *Rhodomela confervoides*. HPLC files of crude extracts of this fungus cultured in both solid rice medium and liquid PDB medium showed a rich diversity (Figure 1), suggesting this fungus can produce abundant secondary metabolites. Chemical investigation of this fungal strain on solid rice media yielded ten structurally diverse polyketides, including two new polyketides, oxalichroman A (**7**) and oxalihexane A (**8**), with strong inhibitory effects on the PATU8988T cell line, as well as eight known compounds, 6,7-dihydroxy-3-methoxy-3-methylphthalide (**9**), chrysoalide B (**10**), rubralide C (**11**), *cis*-(3*RS*,4*SR*)-3,4-dihydro-3,4,8-trihydroxynaphthalen-1(2*H*)-one (**12**), 2,5-dimethyl-7-hydroxychromone (**13**), (7*R*)-(hydroxy(phenyl)methyl)-4*H*-pyran-4-one (**14**), 6-benzyl-4-oxo-1,4-dihydropyridine-3-carboxamide (**15**), and carbonarone A (**16**) (Weng et al., 2022). Using the OSMAC approach (Figure 1), the fungal endophyte *P. oxalicum* 2021CDF-3 was cultivated in PDB media. As a result, six additional indole derivatives, including two new prenylated indole alkaloids asperinamide B (**1**) and peniochroloid B (**5**), and four related derivatives (compounds **2**–**4** and **6**) (Figure 2), were isolated and identified. Based on extensive spectroscopic analysis via HRESIMS, NMR, and TDDFT-ECD calculations, the chemical structures were successfully determined, including the absolute configurations of compounds **1** and **5**. Structurally, compounds **1** and **2** are identified as possessing a 6/6/5/6/6/6/5 heptacyclic scaffold fused with bicyclo[2.2.2]diazaoctane and substituted piperidine. In all of the previously reported prenylated indole alkaloids, the indole-derived unit and the bicyclo[2.2.2]diazaoctane moiety are usually linked through C-2 and C-3 (e.g., malbrancheamide, notoamide R, penicimutamide D, and taichunamide A) or linked to form a spiro system at C-2 (e.g., brevianamide A) or C-3 (e.g., versicolamide B and paraherquamide A). The newly-discovered **1** was characterized to possess a 6/6/5/6/6/6/5 heptacyclic scaffold containing the unusual 3-pyrrolidone dimethylbenzopyran fused to the bicyclo[2.2.2]diazaoctane moiety, hitherto unknown among this kind of compounds. Compounds **3** and **4**, containing a spiroindoxyl and spirooxindole moiety, respectively, represent spiro systems in these prenylated indole alkaloids. Compounds **5** and **6** are characterized as indole derivatives substituted by an isopentene group at C-7. Moreover, cytotoxic effects on the HeLa, HepG2, FADU, and A549 cell lines were also evaluated. The results indicated that **1** strongly inhibited the growth of the FADU cell line. Herein, we describe the isolation, structural determination, and cytotoxic effects of new compounds **1** and **5**.

## 2 Materials and methods

### 2.1 General experimental procedures

Optical rotations were measured with a JASCO P-1020 digital polarimeter (Tokyo, Japan) in MeOH. UV spectra were obtained with a Lambda 35 UV/Vis spectrophotometer (Perkin Elmer, Waltham, USA). ^1^H and ^13^C NMR data were acquired with an Agilent DD2 spectrometer (500 MHz for ^1^H and 125 MHz for ^13^C) (Santa Clara, CA, United States). Chemical shifts (*δ*) are referenced using residual deuterium reagent signals as an internal standard. The 1D NMR assignments were confirmed by the following ^1^H-^1^H COSY, HSQC, and HMBC experiments. HRESIMS spectra were taken with a scientific LTQ Orbitrap XL spectrometer (Thermo Scientific, Waltham, United States). Preparative HPLC separations were conducted with an Agilent 1,260 system. Commercial silica gel (200–300 mesh, Qingdao Marine Chemical Factory, Qingdao, China), octadecylsilyl reversed-phase gel (30–50 μm, YMC Co., Ltd., Japan), and Sephadex LH-20 (GE Healthcare, United States) were purchased and subjected to column chromatography.

### 2.2 Isolation and identification of *P. Oxalicum* 2021CDF-3

The fungus *P. oxalicum* 2021CDF-3 used in this study was previously isolated from the inner tissue of the marine red alga *R. confervoides* and was obtained from Lianyungang, Jiangsu, China. The internal transcribed spacer sequence of 2021CDF-3 displayed 99% identity to that of the reported *P. oxalicum* species. This sequence has been submitted to the GenBank database with no. OP349593. This fungus was preserved at the School of Food and Pharmacy, Zhejiang Ocean University.

### 2.3 Fermentation, extraction, and isolation

Fermentation: Previously, the fungal strain *P. oxalicum* 2021CDF-3 was fermented on solid rice media, which yielded ten structurally diverse polyketides (Weng et al., 2022). To explore the metabolic potential of this strain, the OSMAC approach was used to cultivate this fungus in PDB medium. Mycelia of *P. oxalicum* 2021CDF-3 grown on PDA media (Solarbio Life Sciences Co., Ltd., Beijing, China) were inoculated into a 1 L Erlenmeyer flask containing 300 mL of PDB media (Solarbio Life Sciences Co., Ltd.). Afterwards, the flask was incubated at 28°C at 200 rpm for five days. The whole culture medium was then transferred into 100 × 1 L Erlenmeyer flasks containing PDB medium. Finally, all flasks were fermented statically at 28°C for 30 days.

Extraction: All the culture materials (both the broth and the media) were extracted with EtOAc for three times (each with 30 L EtOAc) at room temperature. A total of 100 L organic solution was obtained. Then, the entire organic solution was concentrated under reduced pressure (−0.1 Mpa) to afford 28.0 g of the EtOAc crude extract. The yield of the EtOAc crude extract was 0.28 g/L.

Isolation: The obtained EtOAc crude extract was partitioned through silica gel VLC and eluted with increasing gradient elution (petroleum ether/EtOAc, from 20:1 to 1:1, v/v, and then CH_2_Cl_2_/MeOH, from 20:1 to 5:1, v/v) to generate eight fractions (A1 − A8). Fraction A5 (1.2 g), which was eluted with petroleum ether/EtOAc 1:1, was separated by an octadecylsilyl reversed-phase silica gel using a stepwise gradient elution of MeOH/H_2_O (from 10 to 100%, v/v) to yield five subfractions (A5.1 − A5.5). Subfraction A5.3, eluted with 40% MeOH/H_2_O, was separated by preparative TLC (CH_2_Cl_2_/acetone 10:1, v/v) to give compound **1** (5.6 mg). Subfraction A5.4, eluted with 50% MeOH/H_2_O, was separated by preparative HPLC (MeOH/H_2_O 65%) to afford compounds **3** (*t*_R_ = 16.2 min, 2.5 mg) and **4** (*t*_R_ = 12.5 min, 4.9 mg). Subfraction A5.5, eluted with 60% MeOH/H_2_O, was separated by Sephadex LH-20 (15 mm × 800 mm, MeOH as elution solvent) to afford compound **2** (1.9 mg). Fraction A6 (2.0 g), which was eluted with CH_2_Cl_2_/MeOH 20:1, was separated by silica gel column chromatography (20 mm × 800 mm, loaded with 200–300 mesh silica gel, CH_2_Cl_2_/MeOH as elution solvent, from 30,1 to 10,1, v/v) to yield compounds **5** (4.4 mg) and **6** (3.8 mg).

Asperinamide B (**1**): colorless oil; [*α*]^20^_D_ + 16.2 (*c* 0.20, MeOH); UV (MeOH) *λ*_max_ (log *ε*) 231 (4.26), 262 (4.27), 390 (3.53); IR (KBr) *ν*_max_ 3,418, 2,940, 2,348, 1,694, 1,594, 1,388, 1,083 cm^−1^; ^1^H and ^13^C NMR data (measured in CD_3_OD) (see Table 1); HRESIMS *m/z* 494.2262 [M + H]^+^ (calcd for C_27_H_32_N_3_O_6_, 494.2291).

**Table 1:** NMR spectroscopic data for compounds 1 and 5 (^1^H at 500 MHz and ^13^C at 125 MHz).

Position	Compound **1**^a^		Position	Compound **5**^b^	
	*δ*_H_ (mult, *J* in Hz)	*δ*_C_, type		*δ*_H_ (mult, *J* in Hz)	*δ*_C_, type
2		93.4, C	1-NH	10.34 (br s)	-
3		197.7, C	2		178.5, C
4	7.33, d (8.4)	124.7, CH	3		74.0, C
5	6.34, d (8.4)	109.4, CH	4	7.15 (m, overlap)	122.3, CH
6		163.2, C	5	6.95 (m)	122.2, CH
7		105.0, C	6	7.14 (m, overlap)	127.6, CH
8		156.5, C	7		128.9, C
9		114.5, C	8		139.9, C
10	6.28, s	73.6, CH	9		132.2, C
11		60.7, C	10		41.0, C
12		168.9, C	11	3.61 (dd, 8.6, 2.3)	79.6, CH
14	3.45, m 3.35, m	43.6, CH_2_	12	3.19 (dd, 16.9, 7.7) 2.88 (dd, 11.0, 8.6)	63.6, CH_2_
15	2.05, m; 1.92, m	24.0, CH_2_	13	1.38 (s)	27.3, CH_3_
16	2.67, m; 1.89, m	28.5, CH_2_	14	1.19 (s)	25.8, CH_3_
17		67.2, C	15	2.19 (m); 2.08 (m)	36.8, CH_2_
18		173.5, C	16	3.95 (m); 3.73 (m)	60.1, CH_2_
20	a: 2.09, m; b: 1.87, m	28.5, CH_2_	18		170.6, C
21	3.07, dd (10.4, 6.0)	41.2, CH	19	1.83 (s)	20.9, CH_3_
22		40.5, C			
23	0.59, s	14.3, CH_3_			
24	1.18, s	19.2, CH_3_			
25	7.00, d (9.9)	118.4, CH			
26	5.77, d (9.9)	127.5, CH			
27		76.5, C			
28	1.54, s	26.9, CH_3_			
29	1.48, s	24.9, CH_3_			
30	3.15, s	50.6, CH_3_			

^a^Measured in CD_3_OD; ^b^Measured in DMSO-d_6_.

Peniochroloid B (**5**): colorless oil; [*α*]^20^_D_ + 115.7 (*c* 0.05, MeOH); UV (MeOH) *λ*_max_ (log *ε*) 214 (3.79), 258 (1.44), 296 (1.35); IR (KBr) *ν*_max_ 3,380, 2,942, 2,831, 1720, 1,454, 1,032, 738 cm^−1^; ^1^H and ^13^C NMR data (measured in DMSO-*d*_6_) (see Table 1); HRESIMS *m/z* 336.1458 [M − H]^−^ (calcd for C_17_H_22_NO_6_, 336.1447).

### 2.4 Computational section

The computational details are shown in Supplementary material.

### 2.5 Cytotoxic assay

The cytotoxicity of isolated compounds **1**–**6** was determined *in vitro* against HeLa, HepG2, FADU, and A549 cells by the CCK8 colorimetric method (Yuan et al., 2020b). Doxorubicin was used as a positive control.

## 3 Results and discussion

### 3.1 Structural elucidation

The EtOAc crude extracts of *P. oxalicum* 2021CDF-3 were initially chromatographed on a silica gel column, and then fractionated by gel chromatography on Sephadex LH-20 to yield the following compounds **1** and **5**.

**Figure 3:**
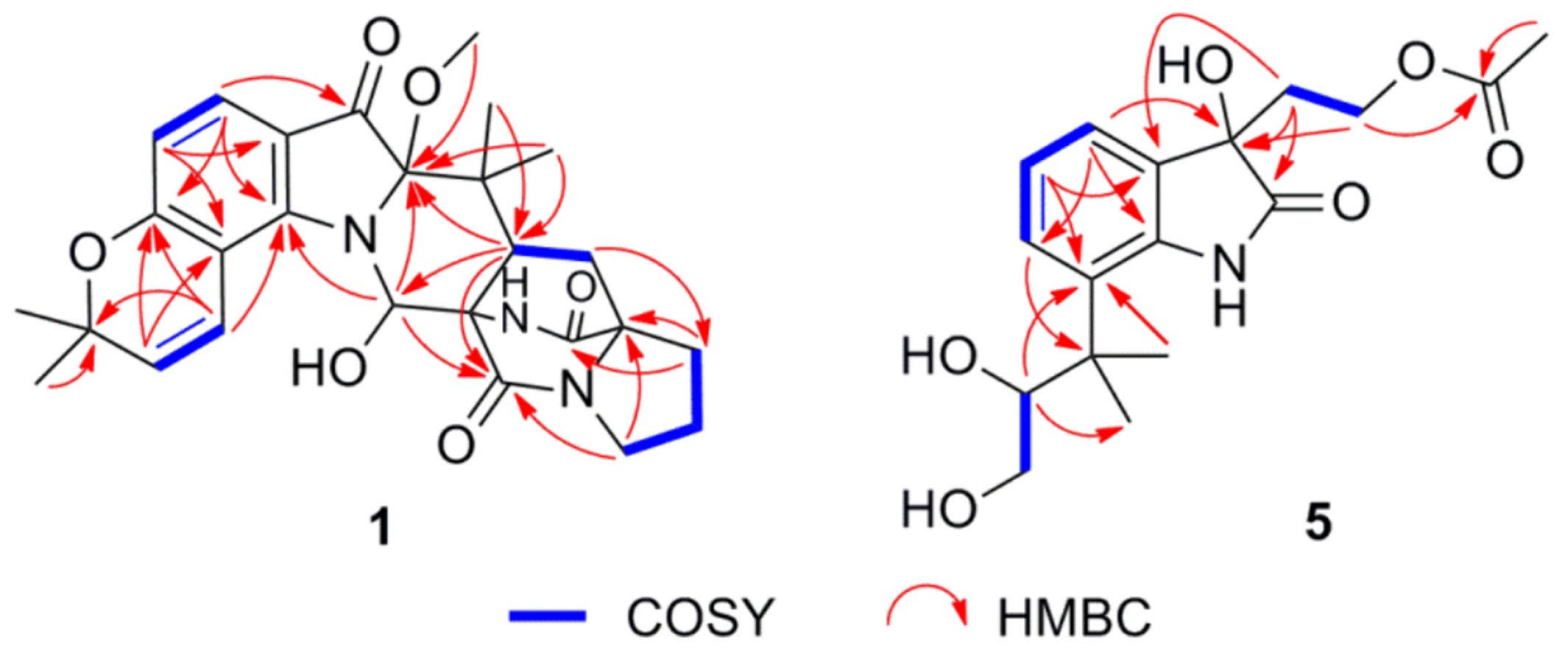
Important ^1^H-^1^H COSY and HMBC correlations of **1** and **5.**

Asperinamide B (**1**) was isolated as a colorless oil (MeOH) and was found to possess the molecular formula of C_27_H_32_N_3_O_6_ according to its HRESIMS data (*m/z* 494.2262 [M + H]^+^, calcd for C_27_H_32_N_3_O_6_, 494.2291). The ^1^H and ^13^C NMR data of **1** (Table 1) revealed similar functional groups to those of asperinamide A (**2**) (Zhao et al., 2023), including three carbonyls [*δ*_C_ 197.7 (C-3), 168.9 (C-12), and 173.5 (C-18)], nine quaternary carbons, six methines [including four sp^2^ at *δ*_H/C_ 7.33 (d, *J* = 8.4 Hz, H-4)/124.7 (C-4), 6.34 (d, *J* = 8.4 Hz, H-5)/109.4 (C-5), 7.00 (d, *J* = 9.9 Hz, H-25)/118.4 (C-25), and 5.77 (d, *J* = 9.9 Hz, H-26)/127.5 (C-26), and an oxygenated sp^3^ at *δ*_H/C_ 6.28 (s, H-10)/73.6 (C-10)], four methylenes [*δ*_C_ 43.6 (C-14), 24.0 (C-15), 28.5 (C-16), and 28.5 (C-20)], and five methyl groups [including one methoxy group at *δ*_H/C_ 3.15 (s, H_3_-30)/50.6 (C-30)]. The NMR data as well as the functional groups of **1** were closely related to those of **2**, indicating that compound **1** was a prenylated indole alkaloid possessing a bicyclo[2.2.2]diazaoctane skeleton. Two carbonyls resonated at *δ*_C_ 168.9 (C-12) and 173.5 (C-18) and two related nitrogenated quaternary carbons resonated at *δ*_C_ 60.7 (C-11) and 67.2 (C-17) convinced this deduction (Kato et al., 2007). Moreover, ^1^H − ^1^H COSY correlations between H_2_-20 and H-21, between H_2_-14, H_2_-15, and H_2_-16, and HMBC correlations (Figure 3) from H_2_-14 to C-12, from H_2_-16 to C-17 and C-18, H_2_-20 to C-16, and from H-21 to C-12, led to the construction of this bicyclo[2.2.2]diazaoctane ring. The remaining substructure of **1** was identical to that of **2**, as confirmed by detailed analysis of the 2D NMR (^1^H − ^1^H COSY and HMBC) data (Figure 3). The abovementioned spectroscopic features confirmed the presence of a 3-pyrrolidone dimethylbenzopyran fused to the bicyclo[2.2.2]diazaoctane moiety. Finally, in the HMBC spectrum of **1**, H_3_-30 showed a correlation to C-2, indicating the location of this methoxy group at C-2 (Figure 3). The gross structure of **1** was therefore assigned and compound **1** was named as asperinamide B. Both asperinamide B (**1**) and asperinamide A (**2**) are identified as possessing a 6/6/5/6/6/6/5 heptacyclic scaffold fused with bicyclo[2.2.2]diazaoctane and substituted piperidine. The main difference between **1** and **2** is that **2** contains an unique pyrano[2,3-*g*]indole, while **1** possesses an indoxyl moiety.

**Figure 4:**
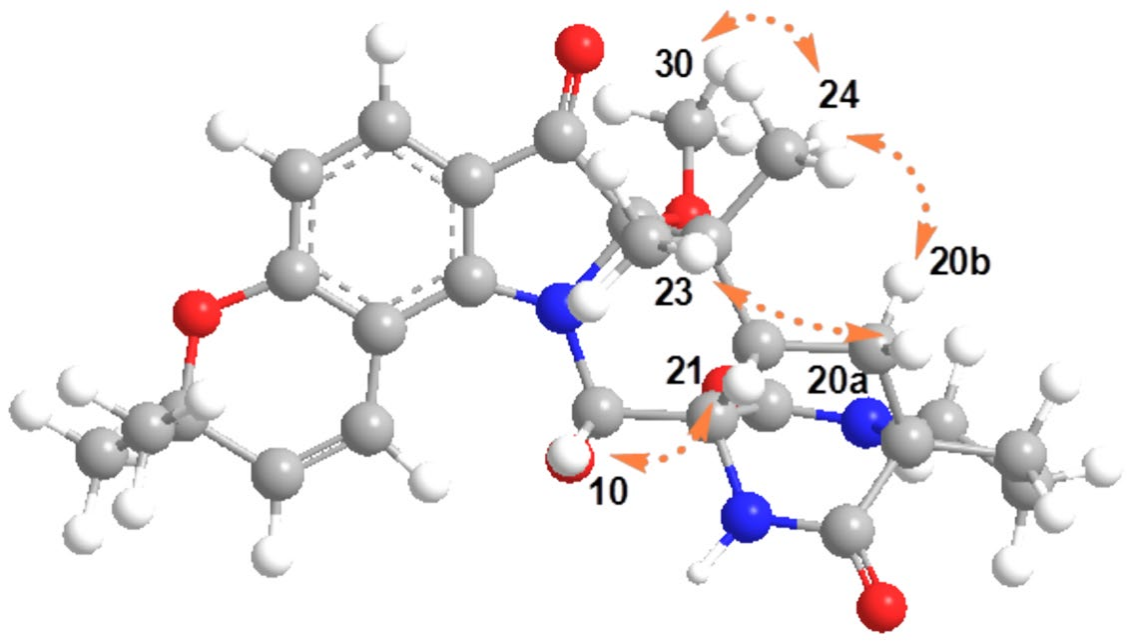
Key NOESY correlations of **1.**

**Figure 5:**
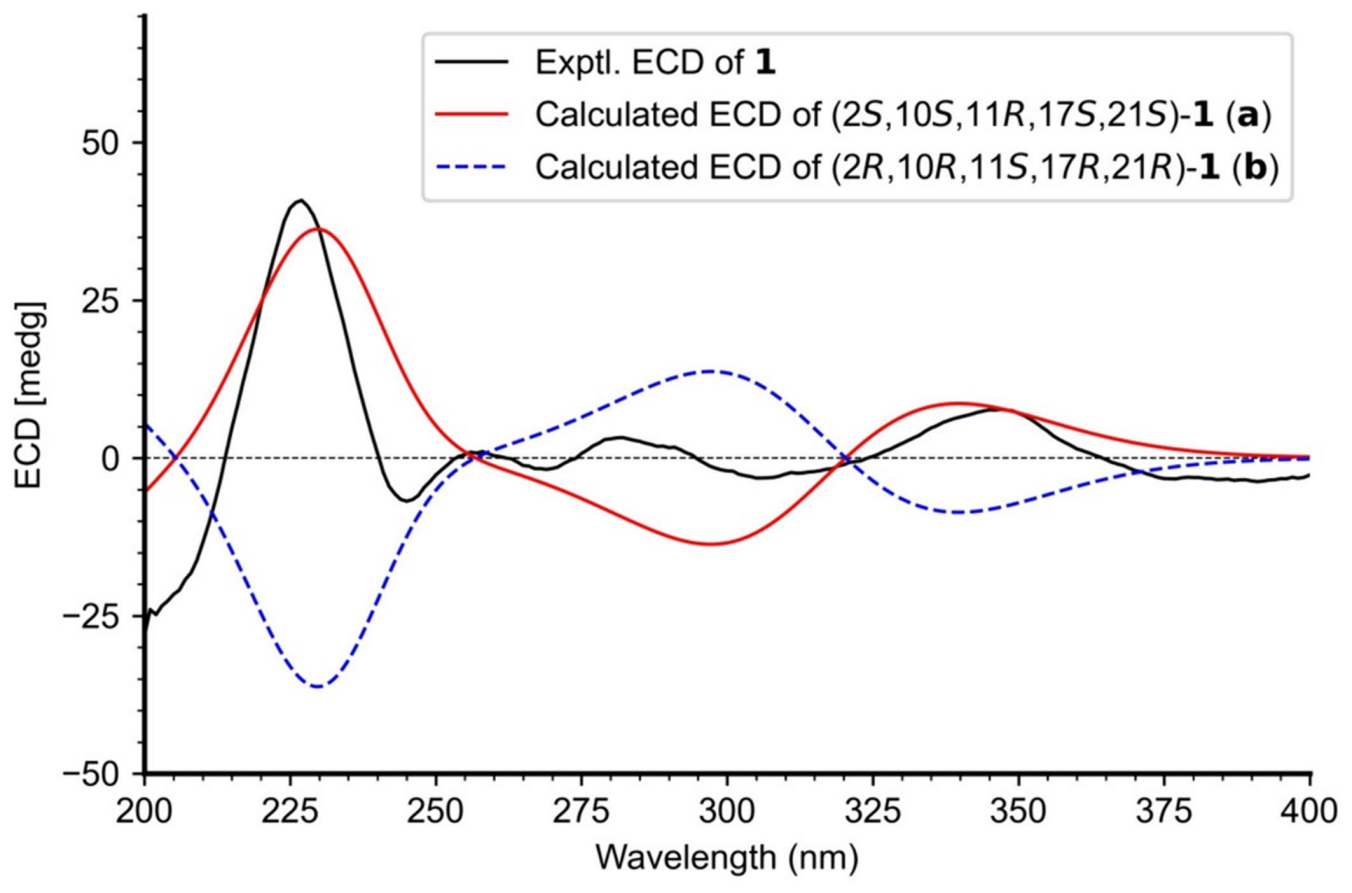
Experimental and calculated ECD spectra of **1.**

**Figure 6:**
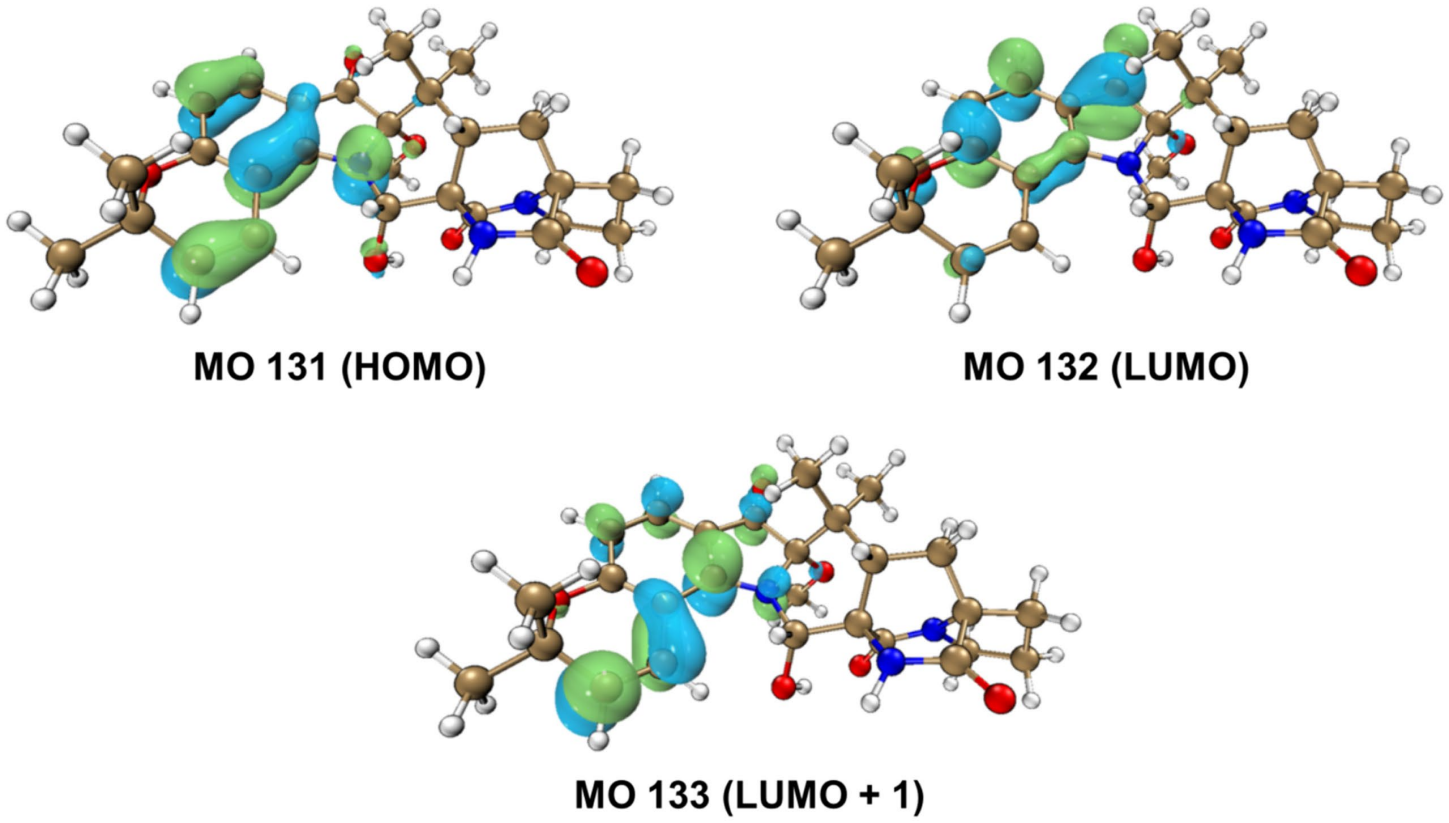
Key molecular orbitals (MOs) involved in the important transitions of **1.**

The relative configurations of **1** were determined based on its NOESY relationships (Figure 4). The observed NOE interactions of H-20a with H_3_-23, and of H-20b with H_3_-24, suggested that H-20a and H_3_-23 were oriented in the same direction, tentatively assigned as β, while H-20b and H_3_-24 were oriented in the α direction. Further, the NOE interactions of H-10/H-21/H_3_-23 indicated that H-10 and H-21 were β-oriented. Similarly, NOE interactions of H_3_-30 with H_3_-24 suggested that H_3_-30 was α-oriented. By comparing the chemical shifts with those of compound **2**, the chemical shifts from C-11 to C-21 were very close, indicating a shared relative configuration. In summary, the relative configuration of compound **1** was determined as 2S*, 10S*, 11R*, 17S*, 21S*. The absolute configurations of **1** were initially determined by comparing its ECD spectrum with those of previously known compounds. Previous studies revealed that the arrestive Cotton effect at 200–250 nm caused by an n-π* transition of the amide bond was responsible for the bicyclo[2.2.2]diazaoctane framework (Kato et al., 2007). The ECD spectrum of **1** displayed a positive Cotton effect at 230 nm, which was similar to that of notoamides (Kato et al., 2007). Therefore, the absolute stereochemistry of **1** was deduced to be 2*S*,10*S*,11*R*,17*S*,21*S*. Meanwhile, compound **1** was also subjected to TDDFT-ECD calculations at the CAM-B3LYP/6-311G(d) level. As expected, the good agreement of the high-energy ECD transitions (Figure 5) allowed the determination of the absolute configuration of **1**. The positive CE at ~230 nm was ascribed to the electron transition from MO131 (HOMO) to MO133 (LUMO +1). The positive CE at ~330 nm was caused by the electron transition from MO131 (HOMO) to MO132 (LUMO) (Figure 6). This approach solidified the determination of the stereochemistry and highlighted the role of ECD spectroscopy and TDDFT calculations in the structural analysis of complex molecules.

Peniochroloid B (**5**) was isolated as a colorless oil (MeOH). Its molecular formula was assigned as C_17_H_23_NO_6_ according to the primary HRESIMS peak generated at *m/z* 336.1458 [M − H]^−^ (calcd for C_17_H_22_NO_6_, 336.1447). The UV peaks at 214, 258, and 296 nm indicated the presence of an indolinone subunit, which was identical to that previously reported for the compound sclerotiamide (Whyte et al., 1996). The ^1^H NMR data of **5** (Table 1) showed three aromatic methines at *δ*_H_ 7.15 (m, H-4), 6.95 (m, H-5), and 7.14 (m, H-6), one oxygenated methine at *δ*_H_ 3.61 (dd, *J* = 8.6, 2.3 Hz, H-11), two oxygenated methylenes at *δ*_H_ 3.19 (dd, *J* = 16.9, 7.7 Hz, H-12α), 2.88 (dd, *J* = 11.0, 8.6 Hz, H-12β), 3.95 (m, H-16α), and 3.73 (m, H-16β), and three methyl groups at *δ*_H_ 1.38 (s, H_3_-13), 1.19 (s, H_3_-14), and 1.83 (s, H_3_-19). The ^13^C NMR spectra of **5** revealed two ester/amide carbonyls at *δ*_C_ 178.5 (C-2) and 170.6 (C-18), four methines including three aromatic at *δ*_C_ 122.3 (C-4), 122.2 (C-5), and 127.6 (C-6), and one oxygenated at *δ*_C_ 79.6 (C-11), three methylenes including two oxygenated at *δ*_C_ 63.6 (C-12) and 60.1 (C-16), three methyls, and five quaternary carbons. Analysis of the UV and NMR data led to the identification of an indolinone framework. The relevant ^1^H and ^13^C NMR data (Table 1) for **5** are consistent with those for peniochroloid A (**6**) (Liu et al., 2023). The presence of an isopentene group was determined by the COSY correlation between H-11 (*δ*_H_ 3.61, dd, *J* = 8.6 and 2.3 Hz) and H_2_-12 (*δ*_H_ 3.19, dd, *J* = 16.9 and 7.7 Hz; 2.88, dd, *J* = 11.0 and 8.6 Hz) and correlative HMBC correlations from H-11 to C-13/C-14 and from H_3_-13 to C-7 (Figure 3), as is found in peniochroloid A. The COSY correlation of the methylene protons at *δ*_H_ 2.19 and 2.08 (H_2_-15) with oxygenated methylene protons at *δ*_H_ 3.95 and 3.73 (H_2_-16), together with additional HMBC correlations observed from H_2_-15 to C-2, C-3, and C-9, indicated that both oxygenated quaternary carbon C-3 and OCH_2_CH_2_-unit methylene C-15 were linked. The remaining methyl proton signal at *δ*_H_ 1.83 (H_3_-19) correlated with the ester carbonyl C-18, requiring the connection of H_3_-19 with C-18 to form the acetyl group and completing the assignment of the planar structure of **5**, as shown in Figure 2.

**Figure 7:**
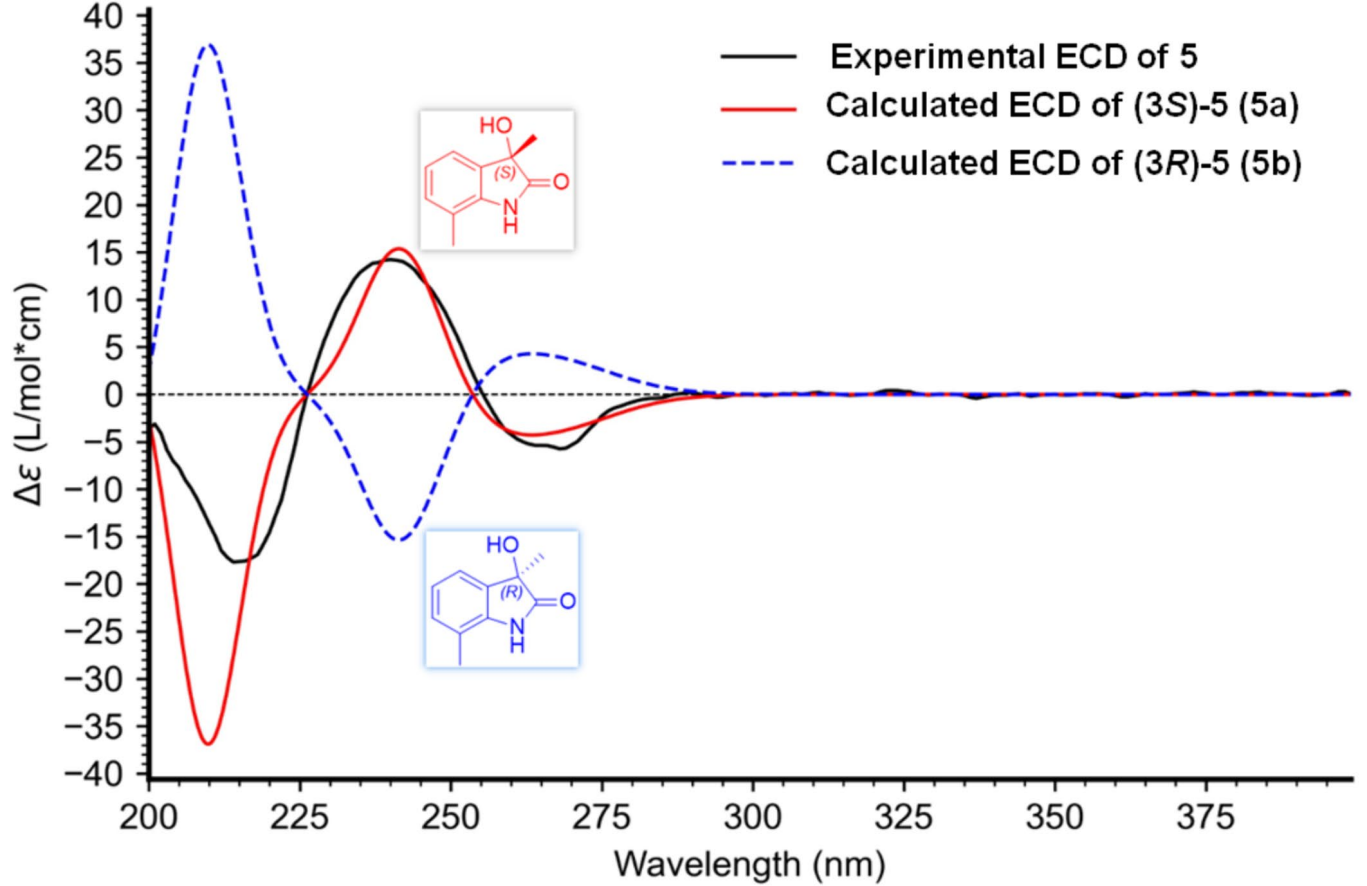
Experimental and calculated ECD spectra of **5.**

Mosher’s method is considered as a useful tool to determine the absolute configuration of C-11 in compound **5**. However, hampered by a deficiency in sample quantity, compound **5** was unable to undergo Mosher’s experiment. The structures of compounds **5** and **6** were closely similar. In their biosynthetic pathways, the segment from C-10 to C-14 was derived from a prenyl group attached to C-7. Compound **6**, a known compound, exhibited an *R* configuration at C-11. A comparison of the chemical shifts for C-10 to C-14 in both compounds revealed their structural similarity and identical chirality at C-11. By carefully comparing the chemical shifts of C-11 and adjacent carbons, and considering that compounds **5** and **6** shared the same biosynthetic pathway (specifically the addition of an isopentenyl unit at C-7 via an isopentenyl transferase), it was inferred that the chirality at C-11 for both compounds should be consistent. The configuration at C-11 in compound **5** was supposed to be *R*. The side chains at positions C-5 and C-7, alongside the side chain at C-3, contributed minimally to the Cotton effects observed in the ECD spectra. Consequently, we simplified these side chains to methyl groups, yielding 3-hydroxy-3,7-dimethylindolin-2-one, featuring a sole chiral center at C-3. By computing the ECD spectra for both the 3*S* and 3*R* configurations and comparing these with the experimental ECD spectra of compound **5**, we were able to definitively ascertain the absolute configuration of C-3 in compound **5**. Upon comparison with the measured spectra (Figure 7), we concluded that the stereochemistry of the C-3 position was *S*.

In addition, four previously reported indole derivatives were finally characterized as asperinamide A (**2**) (Zhao et al., 2023), amoenamide C (**3**) (Zhang et al., 2019), sclerotiamide (**4**) (Whyte et al., 1996), and peniochroloid A (**6**) (Liu et al., 2023), respectively, by comparison of their spectroscopic data with literatures.

### 3.2 Cytotoxic activity

Prenylated indole alkaloids containing the bicyclo[2.2.2]diazaoctane framework have been reported to possess remarkable biological activities, including antitumor, antibacterial, anti-inflammatory, anthelmintic, and insecticidal activities. Based on the same structural characteristics between asperinamide B (**1**) and other this kind of compounds, the new prenylated indole alkaloid **1** was supposed to possess high potential biological significance. The cytotoxicities of compounds **1**–**6** against four human tumor cell lines (HeLa, HepG2, FADU, and A549) were measured. The cells were treated with compounds **1**–**6** at concentrations of 0.001 μM, 0.01 μM, 0.1 μM, 1 μM, 10 μM, 50 μM, 100 μM, and 200 μM for 48 h. The CCK-8 assay results are shown in Table 2. Compound **1** demonstrated dose-dependent cytotoxicity against the human pharyngeal squamous cell line FADU, with an IC_50_ value of 0.43 ± 0.03 μM. This result indicated that the unusual 3-pyrrolidone moiety in **1** may play an important role in cytotoxic activity. Moreover, compound **6** showed higher activity (IC_50_ = 15.30 ± 0.13 μM) against the A549 cell line than compound **5** (IC_50_ = 29.84 ± 0.21 μM), suggesting that the ester carbonyl group in **6** may enhance cytotoxic activity. Head and neck squamous cell carcinoma (HNSCC) is one of the six major malignant tumors worldwide. Hypopharyngeal squamous cell carcinoma (HSCC), accounting for 3 to 5% of all HNSCC cases, has become a current research hotspot due to its high incidence and mortality rates (Chen et al., 2017). Despite significant progress in the treatment of HSCC in recent years, the use of conventional chemotherapy drugs is limited by drug resistance and side effects in tumor drug therapy. Therefore, there is an urgent need to discover alternative antitumor drugs. Compound **1** showed high inhibitory activity against the FADU cell line, comparable to the positive control doxorubicin. Further pharmacological studies will provide evidences to reveal this compound as a potential lead compound for anti-FADU drugs.

**Table 2:** Cytotoxic results of compounds 1–6 against four human tumor cell lines.

Compounds	HeLa	HepG2	FADU	A549
**1**	> 50	> 50	0.43 ± 0.03	> 50
**2**	> 50	> 50	> 50	> 50
**3**	36.54 ± 0.09	> 50	> 50	> 50
**4**	> 50	> 50	> 50	> 50
**5**	> 50	42.62 ± 0.19	> 50	29.84 ± 0.21
**6**	> 50	> 50	> 50	15.30 ± 0.13
Doxorubicin	1.34 ± 0.02	0.88 ± 0.06	0.07 ± 0.01	1.21 ± 0.08

## 4 Conclusion

In summary, continued chemical investigation of the marine red alga-derived endophytic fungus *P. oxalicum* 2021CDF-3 cultured in PDB media yielded six structurally diverse indole derivatives, including two new prenylated indole alkaloids asperinamide B (**1**) and peniochroloid B (**5**). Compound **1** was characterized as possessing an unusual 3-pyrrolidone dimethylbenzopyran fused to the bicyclo[2.2.2]diazaoctane moiety, which was rare in previously reported prenylated indole alkaloids. *In vitro* cytotoxic assays revealed that **1** strongly inhibited the growth of the FADU cell line, indicating that this compound could be a potential lead compound for anti-FADU drugs. This study reported a new prenylated indole alkaloid featuring a 6/6/5/6/6/6/5 heptacyclic scaffold, which added the structural diversity of these kinds of compounds. In addition, the new prenylated indole alkaloid showed promising cytotoxic activity, which will receive more and more attention from natural product chemists for the further pharmacological and biosynthetic/synthetic interests.

## Data availability statement

The original contributions presented in the study are included in the article/Supplementary material, further inquiries can be directed to the corresponding author.

## Author contributions

WS: Writing – original draft, Methodology. LJ: Writing – original draft, Investigation. YZ: Writing – original draft, Methodology, Formal Analysis. LC: Writing – review & editing.
