# Supplementary material for: New cytotoxic indole derivatives with anti-FADU potential produced by the endophytic fungus Penicillium oxalicum 2021CDF-3 through the OSMAC strategy
Source: Front Microbiol. 2024 May 30;15:1400803. doi: 10.3389/fmicb.2024.1400803 (PMC11169714; doi:10.3389/fmicb.2024.1400803)
Supplement: Supplementary file 1 [file Data_Sheet_1.doc]

**Supplementary Material**

# New Cytotoxic Indole Derivatives with Anti-FADU Potential Produced by the Endophytic Fungus Penicillium oxalicum 2021CDF-3 through the OSMAC Strategy

**Wei Song ^1,†^, Lianlian Ji ^2,†^, Yanxia Zhang ^3^, Longhe Cao ^1^,***

^1^Department of Otolaryngology, the Third Affiliated Hospital of Wenzhou Medical University, Zhejiang 325200, China

^2^Department of Pediatrics, the Third Affiliated Hospital of Wenzhou Medical University, Zhejiang 325200, China

^3^Shandong Research Center of Engineering and Technology for Safety Inspection of Food and Drug, Shandong Institute for Food and Drug Control, Jinan 250101, China

***Correspondence:**

Longhe Cao: clh991329@126.com

^†^These authors contributed equally to this work.

**Keywords:** *Penicillium oxalicum*; fungal secondary metabolites; indole derivatives; OSMAC method; cytotoxicity

**Table of Contents**

**Figure S1**. HRESIMS spectrum of compound **1**

**Figure S2**. ^1^H NMR (500 MHz, CD_3_OD) spectrum of compound **1**

**Figure S3**. ^13^C NMR (125 MHz, CD_3_OD) spectrum of compound **1**

**Figure S4**. COSY spectrum of compound **1**

**Figure S5**. HSQC spectrum of compound **1**

**Figure S6**. HMBC spectrum of compound **1**

**Figure S7**. NOESY spectrum of compound **1**

**Figure S8**. HRESIMS spectrum of compound **5**

**Figure S9**. ^1^H NMR (500 MHz, DMSO-*d*_6_) spectrum of compound **5**

**Figure S10**. ^13^C NMR (125 MHz, DMSO-*d*_6_) and DEPT spectra of compound **5**

**Figure S11**. COSY spectrum of compound **5**

**Figure S12**. HSQC spectrum of compound **5**

**Figure S13**. HMBC spectrum of compound **5**

**Figure S14**. IR spectrum of compound **1**

**Figure S15**. UV spectrum of compound **1**

**Figure S16**. IR spectrum of compound **5**

**Figure S17**. UV spectrum of compound **5**

**Computational details**


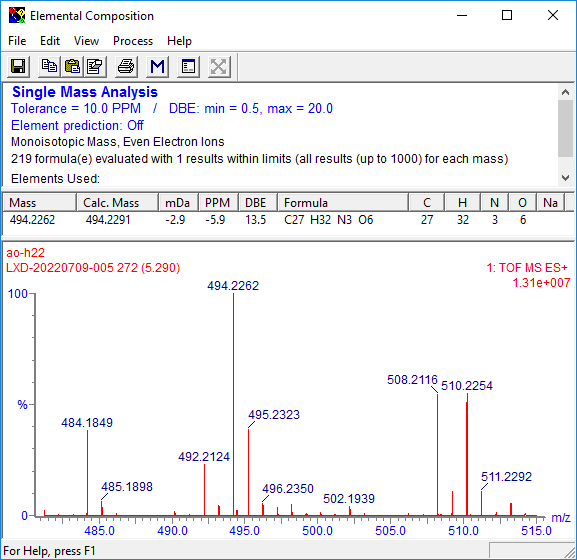


**Figure S1**. HRESIMS spectrum of compound **1**

**Figure S2**. ^1^H NMR (500 MHz, CD_3_OD) spectrum of compound **1**

**Figure S3**. ^13^C NMR (125 MHz, CD_3_OD) spectrum of compound **1**

**Figure S4**. COSY spectrum of compound **1**

**Figure S5**. HSQC spectrum of compound **1**

**Figure S6**. HMBC spectrum of compound **1**

**Figure S7**. NOESY spectrum of compound **1**

**Figure S8**. HRESIMS spectrum of compound **5**

**Figure S9**. ^1^H NMR (500 MHz, DMSO-*d*_6_) spectrum of compound **5**

**Figure S10**. ^13^C NMR (125 MHz, DMSO-*d*_6_) and DEPT spectra of compound **5**

**Figure S11**. COSY spectrum of compound **5**

**Figure S12**. HSQC spectrum of compound **5**

**Figure S13**. HMBC spectrum of compound **5**

**
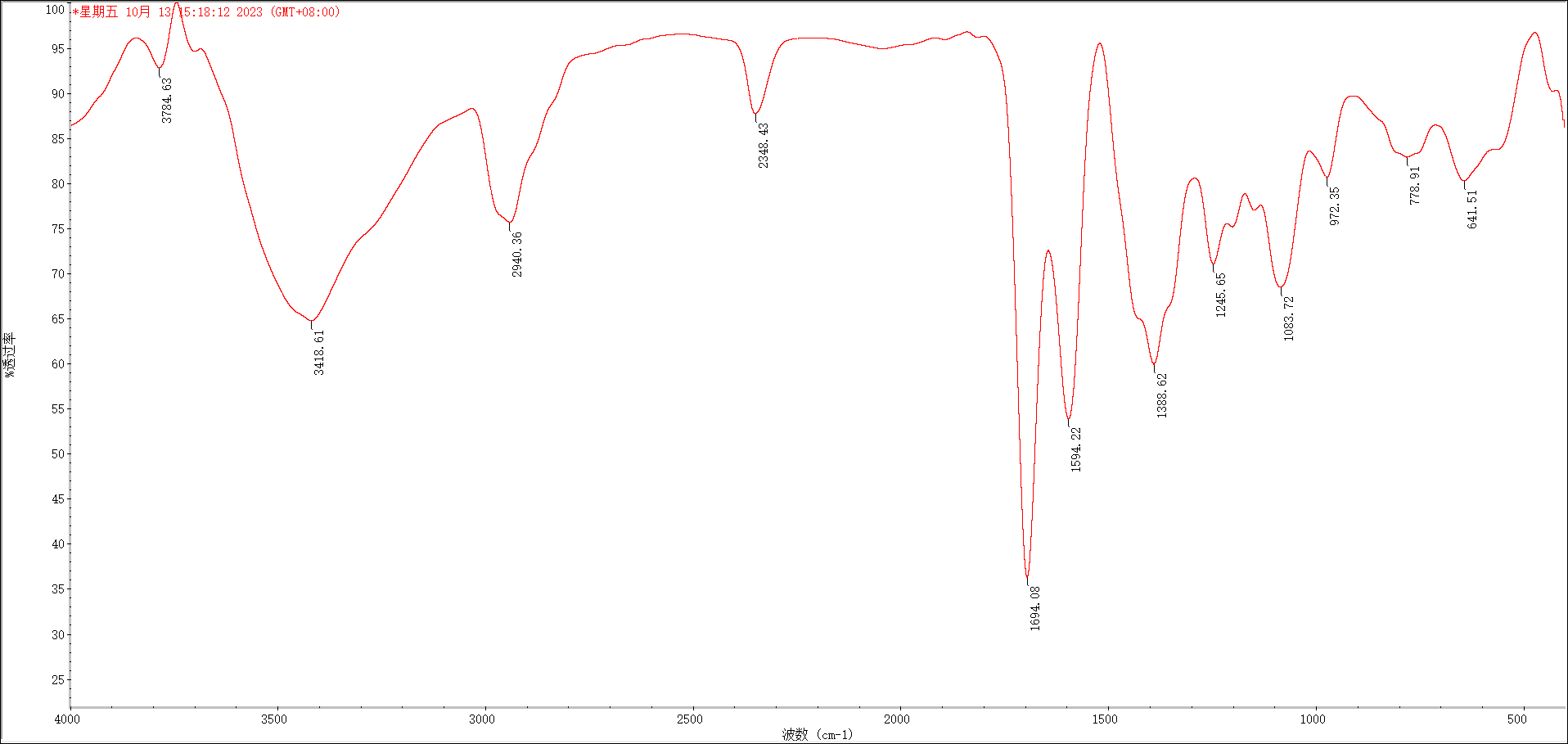
**

**Figure S14**. IR spectrum of compound **1**

**
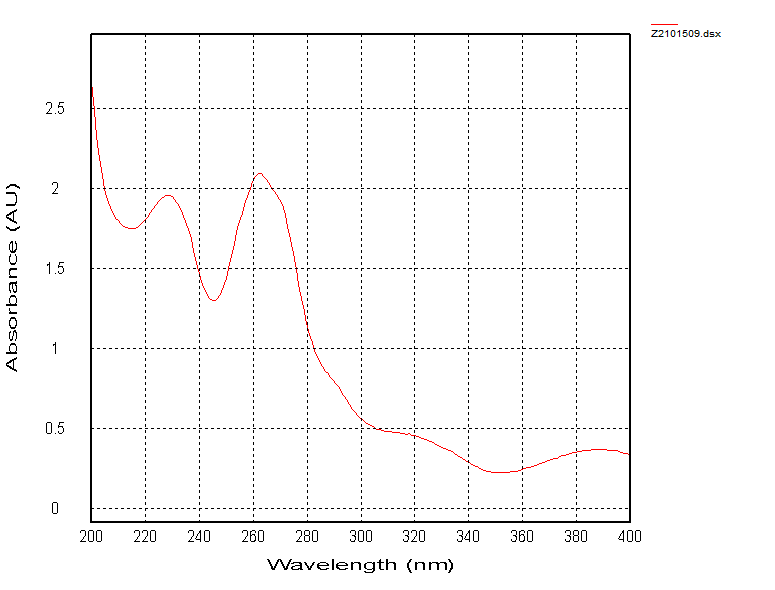
**

**Figure S15**. UV spectrum of compound **1**

**
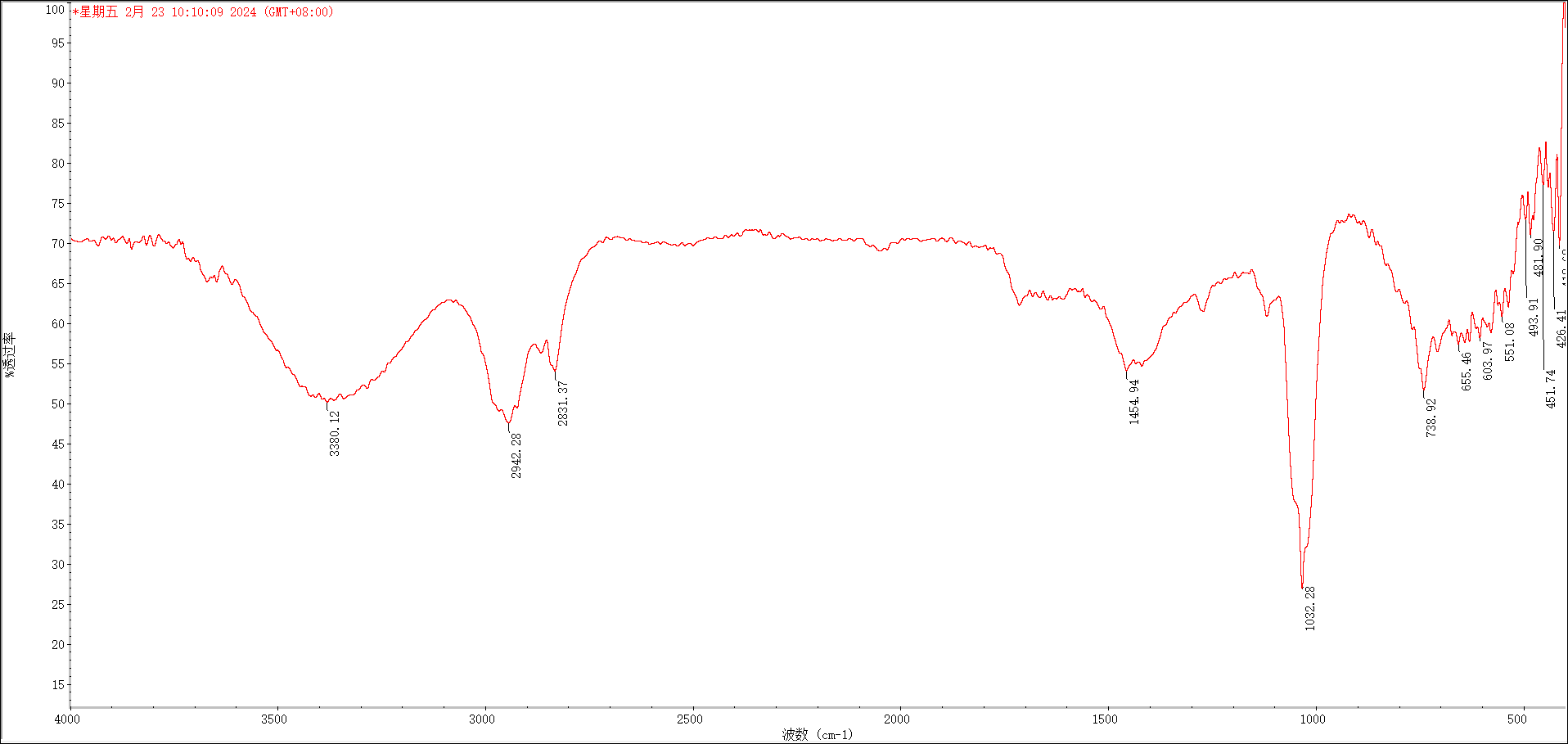
**

**Figure S16**. IR spectrum of compound **5**

**
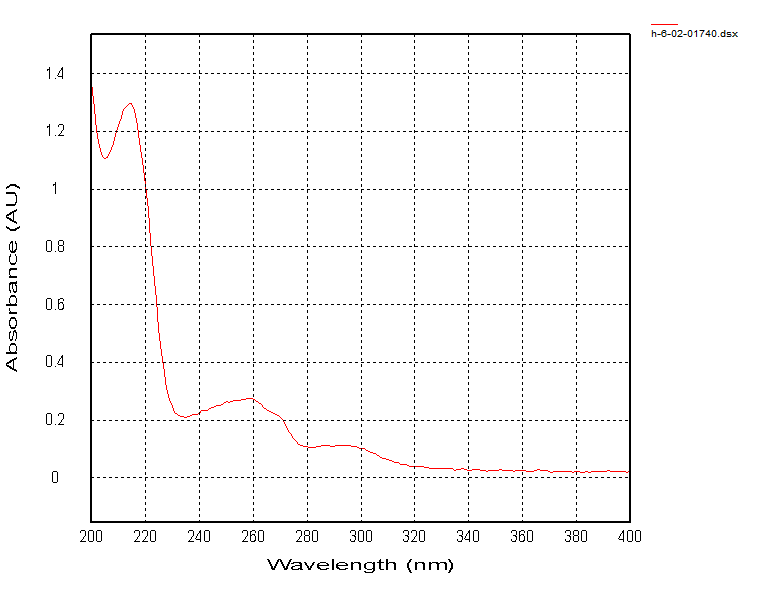
**

**Figure S17**. UV spectrum of compound **5**

**Computational details**

The conformer rotamer ensemble sampling tool (crest) (Pracht et al. 2020; Grimme, 2019) was utilized to generate candidate conformers and DFT calculations were performed using the Gaussian 16 program (Frisch et al. 2016). The conformers within an energy window of 10 kcal/mol were optimized at B3LYP/6-31G(d) level of theory with Grimme's D3 dispersion correction (“EmpiricalDispersion=GD3” key words in input files). Frequency analysis of all optimized conformations was undertaken at the same level of theory to ensure they were true local minima on the potential energy surface. Then, energies of all optimized conformations were evaluated by M062X/6-311+G(2d,p) with D3 dispersion correction. Gibbs free energies of each conformers were calculated by adding “Thermal correction to Gibbs Free Energy” obtained by frequency analysis to electronic energies obtained at M062X/6-311+G(2d,p). Room-temperature (298.15 K) equilibrium populations were calculated according to Boltzmann distribution law. Those conformers accounting for over 2% population were subjected to subsequent calculations.

Time-dependent density-functional theory (TDDFT) ECD calculations were run at CAM-B3LYP/6-311G(d) level of theory in MeOH with IEFPCM solvent model, respectively. For each conformer, 36 excited states were calculated (Pescitelli and Bruhn. 2016). The calculated ECD curves were generated using Multiwfn 3.6 software (Lu and Chen. 2012).

**References**

Frisch, M. J.; Trucks, G. W.; Schlegel, H. B.; Scuseria, G. E.; Robb, M. A.; Cheeseman, J. R.; Scalmani, G.; Barone, V.; Petersson, G. A.; Nakatsuji, H.; Li, X.; Caricato, M.; Marenich, A. V.; Bloino, J.; Janesko, B. G.; Gomperts, R.; Mennucci, B.; Hratchian, H. P.; Ortiz, J. V.; Izmaylov, A. F.; Sonnenberg, J. L.; Williams; Ding, F.; Lipparini, F.; Egidi, F.; Goings, J.; Peng, B.; Petrone, A.; Henderson, T.; Ranasinghe, D.; Zakrzewski, V. G.; Gao, J.; Rega, N.; Zheng, G.; Liang, W.; Hada, M.; Ehara, M.; Toyota, K.; Fukuda, R.; Hasegawa, J.; Ishida, M.; Nakajima, T.; Honda, Y.; Kitao, O.; Nakai, H.; Vreven, T.; Throssell, K.; Montgomery Jr., J. A.; Peralta, J. E.; Ogliaro, F.; Bearpark, M. J.; Heyd, J. J.; Brothers, E. N.; Kudin, K. N.; Staroverov, V. N.; Keith, T. A.; Kobayashi, R.; Normand, J.; Raghavachari, K.; Rendell, A. P.; Burant, J. C.; Iyengar, S. S.; Tomasi, J.; Cossi, M.; Millam, J. M.; Klene, M.; Adamo, C.; Cammi, R.; Ochterski, J. W.; Martin, R. L.; Morokuma, K.; Farkas, O.; Foresman, J. B.; Fox, D. J. *Gaussian 16 Rev. C.01*: Wallingford, CT, 2016.

Grimme, S., Exploration of Chemical Compound, Conformer, and Reaction Space with Meta-Dynamics Simulations Based on Tight-Binding Quantum Chemical Calculations. *Journal of chemical theory and computation* **2019,** *15*, 2847-2862.

Lu, T.; Chen, F., Multiwfn: a multifunctional wavefunction analyzer. *J Comput Chem* **2012,** *33*, 580-92.

Pescitelli, G.; Bruhn, T., Good Computational Practice in the Assignment of Absolute Configurations by TDDFT Calculations of ECD Spectra. *Chirality* **2016,** *28*, 466-74.

Pracht, P.; Bohle, F.; Grimme, S., Automated exploration of the low-energy chemical space with fast quantum chemical methods. *Physical chemistry chemical physics : PCCP* **2020,** *22*, 7169-7192.
